# It’s not you, it’s the design - common problems with patient monitoring reported by anesthesiologists: a mixed qualitative and quantitative study

**DOI:** 10.1186/s12871-019-0757-z

**Published:** 2019-05-28

**Authors:** David W. Tscholl, Lucas Handschin, Julian Rössler, Mona Weiss, Donat R. Spahn, Christoph B. Nöthiger

**Affiliations:** 1 Institute of Anesthesiology, University and University Hospital Zurich, Raemistrasse 100, 8091 Zurich, Switzerland; 2 Department of Management, School of Business and Economics, Free University of Berlin, Garystrasse 21, 14195 Berlin, Germany

**Keywords:** Patient monitoring, Signal processing, Qualitative research, Patient safety, Situation awareness

## Abstract

**Background:**

Patient monitoring is critical for perioperative patient safety as anesthesiologists routinely make crucial therapeutic decisions from the information displayed on patient monitors. Previous research has shown that today’s patient monitoring has room for improvement in areas such as information overload and alarm fatigue. The rationale of this study was to learn more about the problems anesthesiologists face in patient monitoring and to derive improvement suggestions for next-generation patient monitors.

**Methods:**

We conducted a two-center qualitative/quantitative study. Initially, we interviewed 120 anesthesiologists (physicians and nurses) about the topic: common problems with patient monitoring in your daily work. Through deductive and inductive coding, we identified major topics and sub themes from the interviews. In a second step, a field survey, a separate group of 25 anesthesiologists rated their agree- or disagreement with central statements created for all identified major topics.

**Results:**

We identified the following six main topics: 1. “Alarms,” 2. “Artifacts,” 3. “Software,” 4. “Hardware,” 5. “Human Factors,” 6. “System Factors,” and 17 sub themes. The central statements rated for the major topics were: 1. “problems with alarm settings complicate patient monitoring.” (56% agreed) 2. “artifacts complicate the assessment of the situation.” (64% agreed) 3. “information overload makes it difficult to get an overview quickly.” (56% agreed) 4. “problems with cables complicate working with patient monitors.” (92% agreed) 5. “factors related to human performance lead to critical information not being perceived.” (88% agreed) 6. “Switching between monitors from different manufacturers is difficult.” (88% agreed). The ratings of all statements differed significantly from neutral (all *p* < 0.03).

**Conclusion:**

This study provides an overview of the problems anesthesiologists face in patient monitoring. Some of the issues, to our knowledge, were not previously identified as common problems in patient monitoring, e.g., hardware problems (e.g., cable entanglement and worn connectors), human factor aspects (e.g., fatigue and distractions), and systemic factor aspects (e.g., insufficient standardization between manufacturers). An ideal monitor should transfer the relevant patient monitoring information as efficiently as possible, prevent false positive alarms, and use technologies designed to improve the problems in patient monitoring.

**Electronic supplementary material:**

The online version of this article (10.1186/s12871-019-0757-z) contains supplementary material, which is available to authorized users.

## Introduction

The World Health Organization considers continuous patient monitoring during surgical interventions as “extremely important” for patient safety [1].

A patient monitor measures and displays the vital signs of a patient using various sensors and enables care providers to take corrective action if a patient’s vital signs deviate from their normal range. Patient monitoring devices have gained significant relevance in our area of expertise. Anesthesiologists nearly always work directly with a patient monitor.

To perceive the data displayed on a patient monitor and to derive a mental model of the operating room situation, an exchange of information has to take place between the display of the patient monitor and the person interpreting the data shown there [2–8]. The patient monitor serves as the critical interface between the hardware and software components that measure physical quantities in the patient on the one hand and the sensorium and cognition of the human decision makers on the other hand. However, we know from previous research that current standard patient monitoring still has deficits regarding this information transmission. Today’s monitors make use of numbers and curves to transfer vital sign information and display a multitude of individual numerical values and curve forms with very similar ranges of values, e.g., blood pressure, pulse rate and oxygen saturation can all three take a value of 95. Care providers must read all these numbers from the screen one after the other and afterwards cognitively integrate the data to derive meaning, before they can start to establish a complete picture of the patient situation [9–13]. Several research groups have developed innovative technologies, which, at least in theory, were able to communicate a situation overview to users in a faster and easier-to-understand fashion [14–18].

From previous research, we also learned that auditory and visual alarm displays represent a problem in patient monitoring. Alarms are set on the monitor to alert if a vital sign exits its normal range. They are often false positive, e.g., as a result of measurement artefacts, leading to alarm fatigue and potentially causing true positive alarms to go unnoticed because of induced insensitivity [19–24].

The rationale for this study was to learn more about the problems anesthesiologists consider common in their daily work with patient monitors. We hoped that these results would allow us to identify critical aspects for further development in future patient monitors.

## Methods

The Ethics Committee of the Canton of Zurich, Zurich, Switzerland issued a declaration of no objection for this study (Business Administration System for Ethics Committees Req-2016-00103). Additionally, all participants signed an informed consent in which they agreed to the evaluation of their answers for medical research. We did not record audio or video.

### Study design

In planning the study and conducting the analyses, we followed the “Consolidated Criteria for Reporting Qualitative Research” checklist and relevant guidelines for qualitative data analysis and reporting [25].

The study consisted of a qualitative and a quantitative part. First we conducted semi-structured interviews to identify major topics and subthemes from interviews of anesthesia professionals about common problems with patient monitors. Then, for the quantitative part, we derived a central statement for each major theme identified in the interviews and asked a separate group of anesthesiologists to rate their agree- or disagreement with these statements.

#### Study participants

The majority of the participants in this study were anesthesiologists from the anesthesia department of the University Hospital Zurich, a maximum care hospital with around 30,000 surgical procedures per year. One participant came from the anesthesia department of the Kantonsspital Winterthur, Switzerland, a teaching hospital with about 10,000 surgical procedures per year.

In both study steps, all participants were either attending or resident physicians, or nurse anesthetists. All staff physicians held an anesthesia board certification, and all nurse participants had completed their anesthesia sub specialization training. We recruited participants who responded to institutional e-mail invitations and additionally asked colleagues in person to participate according to their personal availability.

Most participants knew the data collectors personally before the study, as they worked in the same departments. We explained the background of the study, namely the development and evaluation of an avatar-based patient monitoring technology, in the invitation e-mails and, when approaching a participant directly, in person.

### Part I: qualitative analysis of interview answers

#### Study setup and data collectors

The interviews were conducted at the end of data collection sessions conducted with the intend to develop a novel avatar-based visualization technology for patient monitoring (Visual Patient technology). The methodology and the results of other studies conducted for this project have been published [17, 18]. Before each interview, participants also completed a personal information survey, e.g., age, sex, previous experience with patient monitors.

Two doctors conducted the interviews. Physician one (CBN) was a senior physician at the Institute of Anesthesiology of the University Hospital Zurich with more than 20 years of clinical anesthesia experience. He works 100% clinically, has completed advanced Good Clinical Practice (GCP) courses, and has considerable experience in patient safety.

The second data collector (LH) was a junior doctor in the second year of his anesthesia residency. During this study, he worked in a 50% clinical and 50% research capacity at the University Hospital Zurich. He has completed entry-level GCP courses at the clinical trials center of the University of Zurich.

#### Description of the interview

We conducted the data collection sessions and interviews in the University Hospital Zurich. The question we asked the participants was: “What are the most common problems with patient monitoring in your daily work?”

The interviewers motivated the participants to answer the questions openly with anything that came to their minds. Otherwise, no prompts or instructions were given. There were no time limits.

As the subjects pared their thoughts, the interviewers typed notes into a Microsoft Word document (Microsoft Corp., Redmond, WA, USA) on an Aspire V15 Nitro laptop computer (ACER, Inc., Taipei, Taiwan).

The transcript was visible to participants during data entry and was provided at the end of the interview for comments and corrections if requested.

#### Analysis

**Fig. 1:**
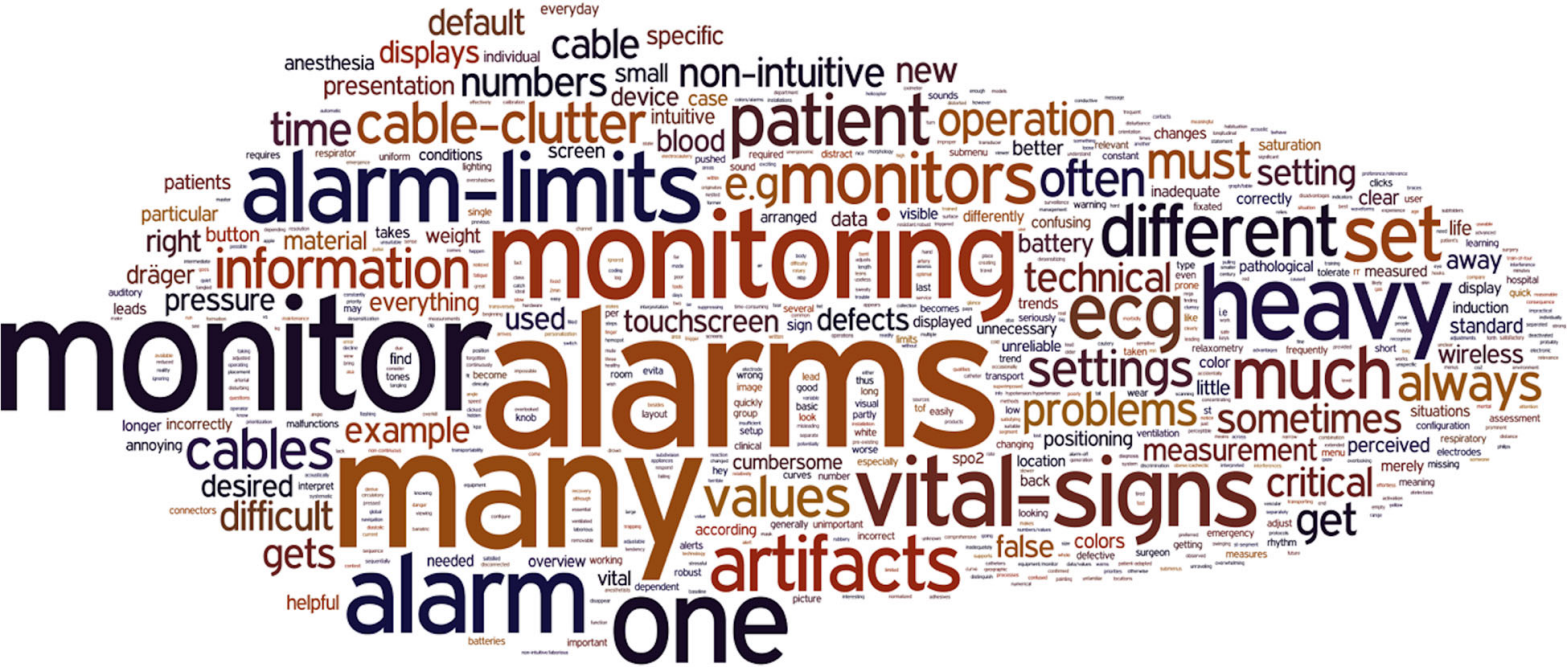
A word cloud created from the participants’ answers to visualize the most common topics. This word cloud was created with Wordle.net. All words were written in lowercase, and commonly occurring English words (e.g., the, is, and, to) were hidden

Before the analysis, we translated the original answers from German to English and unified words of similar meaning to make it easier to count and code words into topics. The matched words were tangling = cable clutter; handling = operation. With the resulting English translation of the answers, we performed a word count (Additional file 1: Table S2) and created a tag cloud (Fig. 1) using Wordle.net. We omitted common English words like ‘and’ or ‘the’ in the word counts and the tag cloud.

Study author DWT, a senior physician, with previous experience in patient safety research, who had not conducted any of the interviews, coded the respondents’ interview answers. Major topics and sub themes were derived from the interview transcripts by applying a two-step process consisting of deductive coding based on word count, followed by an inductive coding process based on the cognitive identification of topics that repeatedly came up in the answers but had not been identified with word counting.

**Fig. 2:**
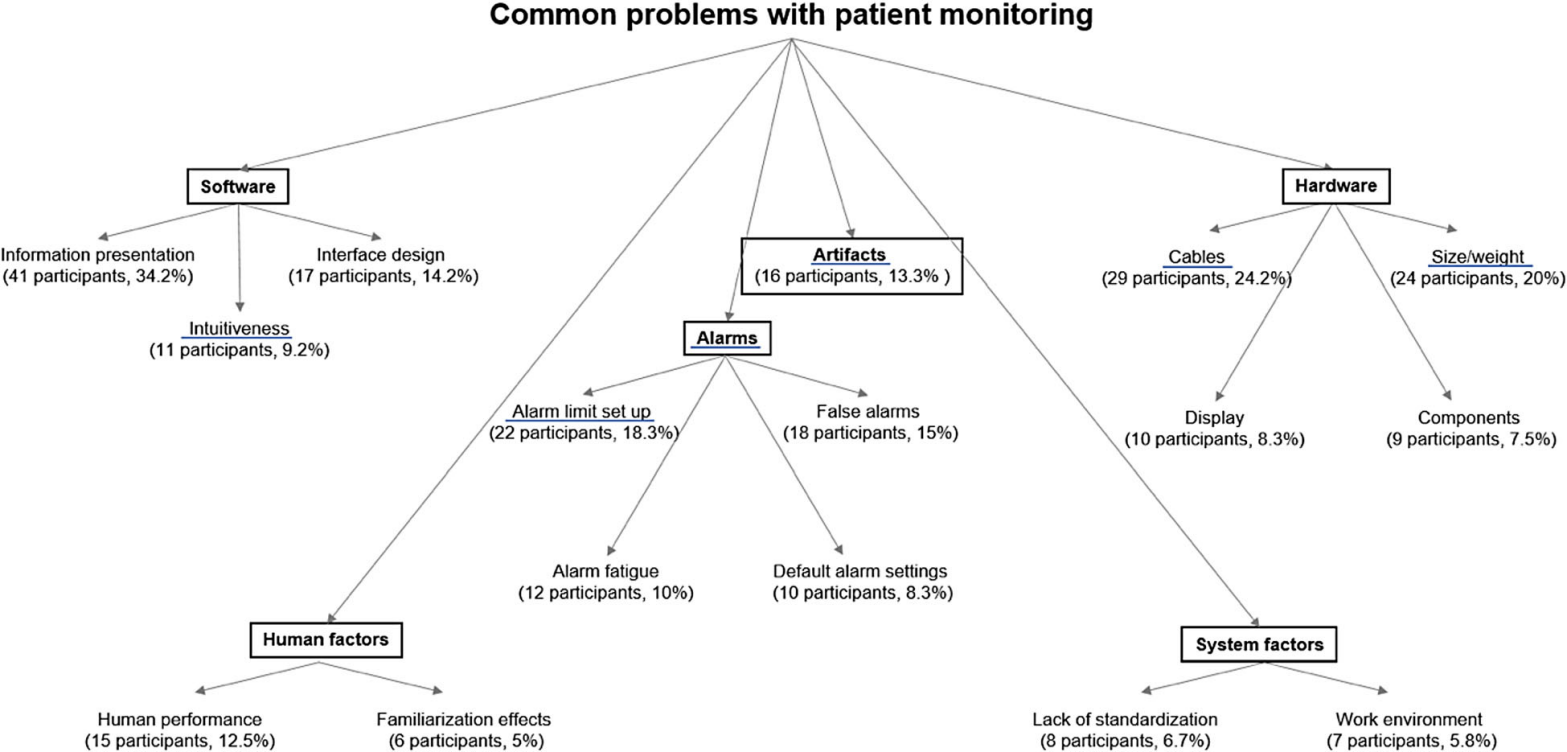
The coding tree with major topics and sub themes, with participant numbers and percentages. We identified the terms underlined in blue by deductive coding based on word counting and the rest by inductive, free coding. We interviewed 120 individual anesthesia experts

We present and discuss these main topics and sub themes with examples in the results and in Table 2. Additionally, we provide a figure of the coding tree (Fig. 2). The complete dataset with unformatted original answers, the stepwise translation, and correction as well as the coding of the answers are provided in Additional file 1: Table S1.

For data management, we used the software Atlas TI 8.0 (Scientific Software Development GmbH, Berlin) and Microsoft Word.

### Part II: quantitative analysis of statement ratings

For the second, quantitative part of this study, we conducted a field survey in which anesthesiologists rated their agree- or disagreement to the qualitative statements.

#### Description of the field survey

In the field survey, we asked the participants to rate a total of six central statements based on the topics identified in the qualitative analysis of the interview responses. Specifically, we created a statement for each of the main theme common problems identified in the interviews. We tried to make these statements relevant for a better understanding of care providers’ problems with patient monitoring.

The statements were evaluated by the participants on five-point Likert scales. The Likert scales consisted of five divisions: 1. “strongly disagree,” 2. “disagree,” 3. “neutral,” 4. “agree,” and 5. “strongly agree.”

#### Statistical analysis

We present the results of the field survey for all six central statements as percentages and as medians with interquartile ranges (IQR). We used the Wilcoxon signed rank test to find out whether the sample medians were significantly different from the neutral rating option. We considered any difference from neutral as practically significant and considered *p*-values of < 0.05 as statistically significant.

Through the participants’ ratings of the central statements, we tried to quantify the agree- or disagreement of the participants with the problems identified. Thereby, internal validity is given to the study.

## Results

### Study and participant characteristics

One hundred and thirty-seven anesthesia professionals participated in the study. For the qualitative part, we evaluated the responses given by 120 individual anesthesia experts. Twenty-five participants took part in the second quantitative part. Eight participants who participated in the interviews also participated in the follow-up study, resulting in a crossing-over between the interview participants and field survey participants of 6%.

The samples in both study steps were gender-, profession-, and experience balanced. Table 1 outlines the study and participant characteristics in full detail.

**Table 1:** Study details and demographic data of study participants. IQR = Interquartile range

	Part I: (participant Interview)	Part II: (field survey)
Duration of data collection in days	248	16
	(April 12th 2016 – December 16th 2016)	(December 5th 2018 – December 21st 2018)
Total number of participants	120	25
Crossing over between parts I and II	8 participants (6%)	
Number of senior physicians (%)	36 (30%)	12 (50%)
Number of residents (%)	45 (37.5%)	12 (50%)
Number of nurse anesthetists (%)	39 (32.5%)	/
Number of female and male participants (%)	57 (47.5%) / 63 (52.5%)	12 (50%) / 12 (50%)
Median (IQR) anesthesia experience group of participants in years	5 to 10 (1 to 5 – more than 10)	6 (2–9.5)
Median (IQR) number of monitoring manufacturers whose products the participants had work experience with:	2 (1–3)	
Number of participants who had experience with the products of the three most common monitor manufacturers in this study:	Dräger (Drägerwerk AG & Co. KGaA. Lübeck, Germany): 120 (100%)	
	Philips (Koninklijke Philips N. V, Amsterdam, The Netherlands): 55 (46%)	
	GE (General Electric Company, Boston, MA, USA): 21 (17.5%)	

As we conducted this study at the University Hospital Zurich, all participants indicated to possess experience with patient monitors of the manufacturer Dräger (Drägerwerk AG & Co. KGaA. Lübeck, Germany), which were in use at the University Hospital Zurich at the time of the study. Additionally, about one of two participants had previous experience with monitors of Philips Healthcare (Koninklijke Philips N. V, Amsterdam, The Netherlands), and about one in six had previous experience with monitors of GE (General Electric Company, Boston, MA, USA).

### Part I: qualitative analysis of interview answers

The word count revealed that the participants most frequently used the following words: alarm = 52, cable = 24, heavy = 20, alarm limits = 19, artifacts = 15, ECG (electrocardiogram) = 15, non-intuitive = 6.

Figure 1 shows the tag cloud created from the words people used in their answers.

Based on the word count and additional inductive, i.e., free coding, we identified the following six main topics with sub themes: 1. “Alarms” with sub themes “alarm limit setup”, “false alarms,” “alarm fatigue,” and “default settings,” 2. “artifacts,” 3. “software” with sub themes “information presentation,” “interface design,” and “intuitiveness,” 4. “hardware” with “cables,” “size/weight,” “display,” and “components,” 5. “human factors” with sub themes “human performance” and “familiarization effects,” and 6. “system factors” with sub themes “lack of standardization” and “work environment.” We describe all major topics and sub themes with participant numbers, percentages, and examples in Table 2 and present the coding tree in Fig. 2.

**Table 2:** The major topics and sub themes with participant counts, percentages and examples. *N* = 120

Major topics	Sub themes	Examples
Alarms	Alarm limit set up	Participant #14: Alarm-limits are set differently by different people - > either the monitor then alerts very quickly or not at all for long.
	(22 participants, 18.3%)	
	False alarms	Participant #9: There are too many false alarms that have no relevance. The ideal would be: If there is no message displayed on the monitor, everything is fine.
	(18 participants, 15%)	
	Alarm fatigue	Participant #48: Frequent false alarms lead to ignoring of alarms.
	(12 participants, 10%)	
	Default settings	Participant #71: Impractical default alarm-limits.
	(10 participants 8.3%)	
Artifacts	16 participants (13.3%)	Participant #12: SpO2 artifacts. Artifacts of the ECG caused by improper positioning of the electrodes.
		Participant #66: Cautery artifacts on the ECG.
		Participant #107: Distinguish artifacts from reality.
Software	Information presentation	Participant #49: Much visual and auditory information, the sense for the relevant gets lost.
	(41 participants, 34.2%)	Participant #102: For a comprehensive state assessment, the gaze must travel across multiple monitors and numbers, which must then be interpreted.
	Interface design	Participant #47: Too many clicks needed to configure the monitor. Great tools hidden in submenus, so they are hard to find.
	(17 participants, 14.2%)	
		Participant #71: In unfamiliar monitors, the patient is effectively worse off due as the vital-signs are perceived much worse and slower.
	Intuitiveness	Participant #23: The screen layout should be easily adjustable (intuitive as Apple products).
	(11 participants, 9.2%)	
		Participant #98: Operation is non-intuitive.
Hardware	Cables	Participant #23: Wireless would be interesting. A wish: a single device on the patient, which measures all vital signs.
	(29 participants, 24.2%)	
		Participant #106: Always cable-clutter.
		Participant #108: Cable disconnected / incorrect values measured.
	Size/weight	Participants #41: Sometimes difficult to transport, smaller transport monitors would be better.
	(24 participants, 20%)	
		Participants #63: Not robust enough for the everyday run (much wear material).
		Participant #110: Patient monitoring is too heavy (kg).
	Display	Participant #36: Small display with reduced resolution. Touchscreen would probably be better or above all more intuitive.
	(10 participants, 8.3%)	
		Participant #46: Numbers too small, not visible from a distance.
	Components	Participant #63: Not robust enough for the everyday run (much wear material).
	(9 participants, 7.5%)	Participant #74: loose contacts...
		Participant #98: Unreliable battery life.
Human factors	Human performance	Participant #82: One pays too little attention to the monitor.
		Participant #82: Although a pathological value is on the monitor, the user does not recognize it because a number is not readily perceptible.
	(15 participants, 12.5%)	
		Participant #95: Tired: One has to look several times until the information arrives.
	Familiarization effects	Participant #13: When changing the hospital or the monitor type, it takes a long time (up to many days) to get used to the new monitors.
	(6 participants, 5%)	
System factors	Lack of standardization	Participant #23: Presentation / standard alarm-limits not uniform.
	(8 participants, 6.7%)	
	Work environment	Participant #97: Lighting conditions and viewing angle to the monitor.
	(7 participants, 5.8%)	Participant #114: A relatively large area with different displays that one must monitor continuously.
Comments/Suggestions	26 participants (21%)	Participant #54: Measured values for non-continuous data collection (e.g., blood pressure) should disappear after a specific time (e.g., 3 or 5 min).
		Participant #116: In emergency situations, one must get a quick and safe overview, which is not always possible with the current monitoring.

### Parent themes

1. **Alarms**

Alarms were a common major common problems topic. Twenty-two interviewees (18%) mentioned alarm limits and alarm configuration as problematic. Other themes included lack of standardization in alarm management, alarm limit settings, e.g., the requirement for different alarm thresholds for different phases of care or different patients.

False alarms were mentioned as problematic by 18 participants (15%). Participants criticized false positive alarms, i.e., a warning when all is ok, and too frequently reoccurring alarms. Twelve (10%) participants specifically used the term “alarm fatigue” and mentioned the danger of desensitization, which may cause a critical patient status to go unnoticed. Participants talked about the sound characteristics of the audio alarms and the problem of discerning which of two or more alerts is more important.

2. **Artifacts**

Artifacts were mentioned as problematic by 16 anesthesiologists (13%). The participants mentioned interactions in measurement such as electrocautery and the ECG or the oxygen saturation probe and patient movement, and the problems of distinguishing artifacts from real problems. One participant suggested that, similar to alarm fatigue, frequent artifacts can lead to ignoring of this sensor and, thereby, losing it for informed decision making. With problems caused by the empty invasive arterial blood pressure measurement sodium chloride infusion bag, another example of a frequently occurring artifact was named.

3. **Software**

Forty-three (34%) interviewees provided problems regarding the presentation of information. The participants complained that they consider the display of information in current monitoring confusing, e.g., the information is number-coded, and interpretation requires skills. The participants reported that they often feel overloaded with information. In addition, participants complained about a lack of standardization in the design and presentation of monitoring information between different manufacturers and hospitals. Eighteen participants (14%) mentioned software interface design aspects, e.g., a lack of intuitiveness in interface designs and the importance of customizability.

4. **Hardware**

Of the 120 total participants, 29 (24%) mentioned problems with cables. On one hand, problems with the technical aspects of cables, e.g., unplugged cables, bent or worn out plugs, ECG electrodes coming off with certain patient conditions, e.g., wet skin or a special patient positioning. On the other hand, the answers included examples of situations in which working with cables is most bothersome, e.g., when changing the patient’s position or transferring a patient. Participants mentioned the large amount of individual cables, the length of the cables and constant entanglements as problematic and wished for a wireless solution.

Twenty-four (20%) participants addressed weight and industrial design issues of the monitoring devices, e.g., bulkiness. Ten (8%) participants complained about the technical characteristics of the displays. They wished for monitoring displays with these properties: “removable,” “large format,” “touchscreen,” “high-resolution”, “vibrant.” Nine participants (8%) mentioned problems with individual components of the monitors. Mainly issues with battery life and built quality of parts, e.g., connectors.

5. **Human factors**

The human factors main topic included human performance and habituation effects, which were named by 15 (13%) and six subjects (5%), respectively. The human factors aspects included problems of interpretation of vital data in a numerical and wave-form presentation and problems with information overload caused by our limited and fatigue sensitive working memory. Participants described habituation effects as substantial and considered switching between different monitoring manufacturers difficult.

6. **System factors**

The anesthesiologists complained about insufficient standardization in monitoring - in both, the hospital and the global healthcare context. Unfavorably mounted monitors, poor operating room lighting conditions, the requirement for a large number of individual monitors, e.g. respirator, patient monitor, syringe pumps, to capture the situation were all mentioned as problematic. A few participants mentioned the poor transport capability of monitors as bothersome.

#### Comments/suggestions

Twenty-six participants (21%) provided ideas that did not fit into any of the major topics outlined above. These included aspects relevant for safety design, e.g., the wish of an anesthesiologist that trend images should always be visible and slow changes over time should be made recognizable for the care provider, or that the alarm off button should only mute the alarm for which it is pressed. Alarm tones should be made more explicit. One participant mentioned that despite all monitoring, we should never forget to look at the patient and the monitors: “Treat the patient, not the monitor.”

### Part II: quantitative analysis of statements rated in an online survey

**Fig. 3:**
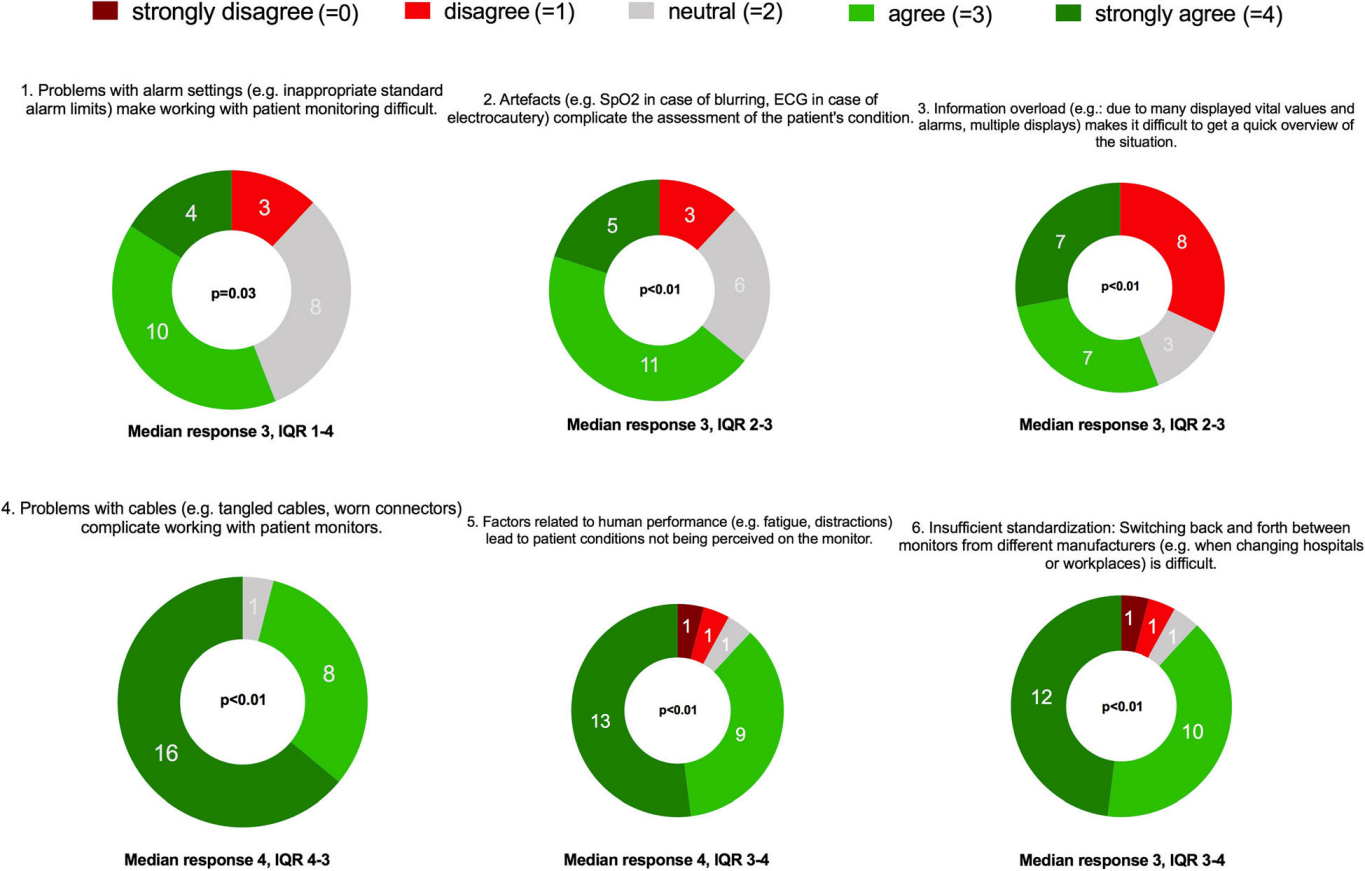
Presentation of the results of the field survey as donut charts with the number of participants who chose a particular category. We used the Wilcoxon signed rank test to evaluate whether the sample medians were significantly different from neutral. IQR = Interquartile range. 0 = strongly disagree, 1 = disagree, 2 = neutral, 3 = agree, 4 = strongly agree. *N* = 25

Figure 3 shows the results of the ratings of the six central statements derived from the qualitative analysis (study part I). The sample medians of all six statements statistically significantly differed from neutral.

## Discussion

**Fig. 4:**
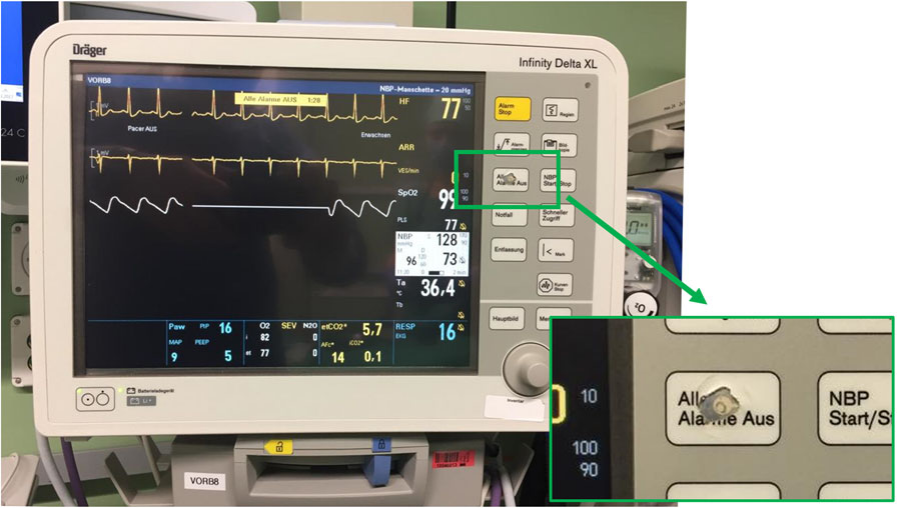
The fact that too many alarms are a common problem is illustrated by the fact that the most worn button on our patient monitors is the “all alarms off” button

In this qualitative and quantitative study, we analyzed the responses that 120 individual anesthesia experts gave in semi-structured interviews to an open-ended interview question asking them what problems commonly occur in their daily work with patient monitors. We then extracted central statements from the interviews and validated them in a quantitative approach through confirmation by a new group of 25 anesthesia professionals. All six statements were significantly agreed on, which by quantitative confirmation of the agreement with the central statements, increases the validity of the results. Although alarm fatigue [19–22], information overload [23], and measurement artifacts [24], had already been identified as problematic, this study confirms that these issues are still regarded as problematic by anesthesiologists in 2018 and, additionally, there still seems to be considerable need for improvement also in previously less-known areas of ubiquitously used patient monitors. As a descriptive example, in Fig. 4, we provide a photo, which illustrates the problem of “too many alarms” and the users’ response to it. In our monitors, the most worn out button is the “all alarms off” button.

Through the evaluation of problems with current monitors, we found answers to the fascinating question: “What properties would an ideal patient monitor have?” The answer to this question could be particularly interesting for virtual and augmented reality head mounted devices of the future, as the desirable characteristics could be tested and implement there easily because it would merely be virtual and not physical, as todays patient monitors.

Based on the responses, an ideal patient monitor would be a high-quality built, lightweight and compact, but still robust device, with a large, high resolution, non-reflecting touchscreen display. It would feature intuitive controls and large, vibrant and colorful, easy to understand visual display of the patient’s situation presenting integrated information from all sensors. It would be resistant to artifacts and only issue an alarm in case of a true positive event. Data transmission, maybe even sensing, would be wireless and immediate. This system would do without cables. Work ergonomics would be excellent, and an international standardization for the appearance of monitoring data would be in effect. The hardware and software components, e.g., display, sensors, cables, interface design, and information presentation of an ideal monitor would make it easy for users to preserve both mental and physical energy, and, thereby, be able to maintain their alertness and performance longer and easier than with today’s state-of-the-art.

In our quantitative analysis 88% of the anesthesiologists agreed to the central statement that factors related to human performance (e.g. fatigue, distractions) can lead to important information being overlooked. It is well known that preserving mental and physical energy of care providers is safety critical, because fatigue (physical, alarm- and other kinds of fatigue) causes errors, which may ultimately lead to patient harm [26–29].

These aspects are important for the heterogenous group of patient monitoring users in healthcare, for example, non-experts, trainees, intensivists, emergency care providers, general ward nurses, paramedics, and tele monitoring, and consumer health users.

There is still much room left for improvement in patient monitoring systems and, based on this study and previous research, we claim that standardization [30], easy understanding [3, 9, 10, 17, 18], and fatigue prevention [31–37] should be sought after to reduce the risk of accidents to happen and improve patient safety and operator well-being in the perioperative area. Policy makers and creators of patient monitors should feel encouraged to satisfy these needs.

### Limitations

The first part of the study has the inherent limitations of interview-based qualitative research. The qualitative analysis gives a complete, detailed description. There is no attempt to assign frequencies to the features identified in the data. Rare phenomena receive the same attention as more common phenomena. The main drawback of a qualitative analysis is that its results cannot be transferred to broader populations with the same certainty as quantitative results. This is because the research results are not checked to see if they are statistically significant or random. [38]

However, initially using a qualitative approach in our study context has yielded many benefits. We were able to identify everyday problems that caregivers have with traditional patient monitoring systems and develop a deeper understanding of the causes of these problems. The results contribute to a better understanding of patient monitoring and provide clues for its further improvement. In the first part of the study, our goal was to explore the full range of problems in patient monitoring without weighing these issues quantitatively. Since the participants in this study were asked to describe the problems, they felt were relevant, the analysis likely truly identified problems that the participants experience when working with patient monitoring and, furthermore, we were able to improve the validity of the central statements through their quantitative rating [39, 40].

Finally, this study was conducted in a small area of Central Europe in an environment with a high standard of care. The work values and thus the user perceived problems may vary in different parts of the world.

## Conclusions

To our knowledge, this study is the first to provide a global overview of the problems anesthesiologists commonly experience when working with patient monitors in real-life patient care. This study confirmed findings of previous research that anesthetists are inconvenienced by inadequate alarms, artifacts, and information overload. Some of the new aspects discovered by this study included the findings that anesthesiologists feel bothered by problems related to monitoring cables and that they considered human factor and systemic factor aspects as relevant in patient monitoring. Human factor aspects included fatigue and distraction, which participants indicated may cause patient monitoring information to not be perceived and systemic factor aspects, which included difficulties in switching back and forth between monitors of different manufacturers. Furthermore, this study enabled us to draft a hypothetical prototype of an ideal monitor. An ideal monitor should have an alarm system that reduces false positive alarms to a minimum, works with as few cables as possible and transfer relevant information easily and quickly and, thereby, make it easier for participants to preserve both mental and physical energy, and maintain their alertness and performance longer and easier. These characteristics of a patient monitor would help keep patient safety and user satisfaction high. Further research should focus on the development of new monitoring technologies to provide the necessary information to care providers as quickly and efficiently as possible. Such developments should undergo extensive testing in realistic simulators before being carefully applied in real patients.

## Additional file

Additional file 1: **Table S1.** The complete dataset, language translation and encoding of participants’ answers to the question “What are the most common problems with patient monitoring in your daily work?” Columns two and three are presented in their original form to allow traceability and may, therefore, contain typos and syntax errors. In column four, “Adjusted English translation,” the Google translation (Alphabet Inc., Mountainview, CA, USA) was adjusted by hand for meaning and syntax using Grammarly (Grammarly Inc., San Francisco, CA, USA). In column 4, we matched words with comparable meaning to facilitate word counting and coding. The matched words were tangling = cable-clutter; not intuitive = non-intuitive; use, handling = operation, parameters = vital-signs; weight, heavy = heavy weight. **Table S2.** The results of the word count with all words that have occurred more than five times. The dark gray shading displays the topics identified by the word count. Irrespective of singular or plural, uppercase or lowercase, the word count of these words was: alarm = 52, cable = 24, heavy = 20, alarm limits = 19, artifacts = 15, ECG = 15, non-intuitive = 6. ECG = electrocardiogram. (DOCX 94 kb)

## Abbreviations

ECG: Electrocardiogram
GCP: Good Clinical Practice
IQR: Interquartile Range
