# Supplementary material for: It’s not you, it’s the design - common problems with patient monitoring reported by anesthesiologists: a mixed qualitative and quantitative study
Source: BMC Anesthesiol. 2019 May 28;19:87. doi: 10.1186/s12871-019-0757-z (PMC6540409; doi:10.1186/s12871-019-0757-z)
Supplement: Supplementary file 1 — Table S1. The complete dataset, language translation and encoding of participants’ answers to the question “What are the most common problems with patient monitoring in your daily work?” Columns two and three are presented in their original form to allow traceability and may, therefore, contain typos and syntax errors. In column four, “Adjusted English translation,” the Google translation (Alphabet Inc., Mountainview, CA, USA) was adjusted by hand for meaning and syntax using Grammarly (Grammarly Inc., San Francisco, CA, USA). In column 4, we matched words with comparable meaning to facilitate word counting and coding. The matched words were tangling = cable-clutter; not intuitive = non-intuitive; use, handling = operation, parameters = vital-signs; weight, heavy = heavy weight. Table S2. The results of the word count with all words that have occurred more than five times. The dark gray shading displays the topics identified by the word count. Irrespective of singular or plural, uppercase or lowercase, the word count of these words was: alarm = 52, cable = 24, heavy = 20, alarm limits = 19, artifacts = 15, ECG = 15, non-intuitive = 6. ECG = electrocardiogram. (DOCX 94 kb) [file 12871_2019_757_MOESM1_ESM.docx]

Supplementary material for manuscript:

**It’s not you, it’s the design - common problems with patient monitoring reported by anesthesiologists: a mixed qualitative and quantitative study.**

**Supplementary Table 1:** The complete dataset, language translation and encoding of participants' answers to the question "What are the most common problems with patient monitoring in your daily work?" Columns two and three are presented in their original form to allow traceability and may, therefore, contain typos and syntax errors. In column four, "Adjusted English translation," the Google translation (Alphabet Inc., Mountainview, CA, USA) was adjusted by hand for meaning and syntax using Grammarly (Grammarly Inc., San Francisco, CA, USA). In column 4, we matched words with comparable meaning to facilitate word counting and coding. The matched words were tangling = cable-clutter; not intuitive = non-intuitive; use, handling = operation, parameters = vital-signs; weight, heavy = heavy weight.

|  | Original, unaltered German answer | Adjusted English translation | Coding |
| --- | --- | --- | --- |
| 1 | Einrichten des Monitorings bei speziellen Fragen.  Die richtige Kurve an den richtigen Ort zu bringen. | Setting the monitoring up for specific questions.  To bring the right curve to the right place. | - **Software:** interface design |
| 2 | Automatische Einstellungen (z.B. Range) ist manchmal gut, manchmal nicht. | Automatic adjustments (e.g., range) are sometimes right, sometimes not. | - **Software:** interface design |
| 3 | Fehlende Alarmbewirtschaftung. zB Unterschiedliche Alarmaktivierung bei Einleitung Aufrechterhaltung und Ausleitung einer Anästhesie. | Lack of alarm management. E.g., different alarm activation (required) for induction, maintenance, and emergence from anesthesia. | - **Alarms:** default settings - **Alarms:** alarm limit set up |
| 4 | Aufwändiges Einstellen der gewünschten Parameter nach Präferenz/Relevanz | Setting up desired vital-signs according to preference/relevance is cumbersome. | - **Software:** interface design |
| 5 | Monitoring ist nicht immer intuitiv.  Man gewöhnt sich sehr an ein bestimmtes Monitoring (mit allen Vor- und Nachteilen) ist aber beispielsweise bei geänderten Farben / Alarmtönen bereits irritiert.  Trendverlauf sollte eigentlich als Default programmiert sein, wenn man einen Saal übernimmt/ablöst wäre es hilfreich zu wissen, was die Baseline eines Patienten war (wird in Zukunft bei elektronischen Anästhesieprotokollen noch wichtiger).  Man ist sehr stark auf einen bestimmten Monitor «fixiert» und hat Mühe sich auf einem neuen Gerät zurechtzufinden. | Monitoring is not always intuitive.  One gets very used to a specific monitor (with all its advantages and disadvantages). For example, one gets confused by different colors/alarms (on a new monitor).  Trends should be visible by default. When taking over a case, it would be helpful to know what the patient's baseline was (this will become even more critical with electronic anesthesia protocols in the future).  One gets used to a particular monitor. Working with a new device is difficult. | - **Software/Hardware:** intuitiveness - **Human factors:** familiarization effects - **Comment/suggestion** |
| 6 | Artefakte. Verunmöglichen Interpretation. «Wo bin ich eigentlich?» | Artifacts. Make interpretation impossible. "What is going on here?" | - **Artifacts** |
| 7 | Unübersichtlich. Zu viele Informationen. Lichtverhältnisse am Monitor schlecht. Position des Moitoring nicht befriedigend (Pendel).  Man ist sehr stark auf einen bestimmten Monitor «fixiert» und hat Mühe sich auf einem neuen Gerät zurechtzufinden. | Confusing. Too much information provided.  Lighting conditions on the monitor terrible.  Installation of the monitor is not satisfying (it is swinging back and forth).  One becomes fixated on a particular monitor, and it is difficult to switch to a new device. | - **Software:** information presentation - **Hardware:** display - **System factors:** work environment - **Human factors:** familiarization effects |
| 8 | «Malen nach Zahlen»: d.h. Orientierung an nichts sagenden Nummern und nicht an Trendgeschehen im Kontext.  Starke Gewöhnung an Reihenfolge, Farbe, Töne, die bei einer Umstellung der Monitore nicht mehr «verhebt».  Viele unwichtige Alarmierungen «übertönen» den einen wichtigen Alarm.  Kabelstecker die verbogen sind, und dadurch nicht mehr passen lästig je schneller es gehen muss  Neue EKG anschlüsse die klammern verhalten sich wie angelhacken, dadurch hohe gefahr gefässzugänge etc. Versehentlich gezogen werden genauso deren gumige oberfläche die ein verheddern verstärkt unterstütz, un ein entwirren sehr zeitaufwendig macht | «Painting by numbers»: i.e., orientation according to unspecific numbers and not according to trends in context. Strong habituation to sequence (of vital sign presentation), colors, auditory displays, which are no longer useable when changing the monitors.  Many unimportant alerts "drown out" the relevant alarm.  Cable connectors that are bent, and thus no longer fit. The more time critical, the more annoying this becomes.  The braces of the new ECG connectors behave like hooks. Thus, there is the danger of accidentally pulling out vascular catheters or other installations. Their rubbery surface supports tangling and makes unraveling very time-consuming. | - **Software:** information presentation - **Human factors:** familiarization effects - **Alarms:** false alarms - **Hardware: cables** (ECG, connections) |
| 9 | Zuviele Fehlalarme «ohne Konsequenz». Gewünscht ist/Idealfall: Falls keine Meldung=alles in Ordnung . | There are too many false alarms that have no relevance. The ideal would be: If there is no message displayed on the monitor, everything is fine. | - **Alarms:** false alarms - **Comment/suggestion** |
| 10 | Defekte. Materialverschleiss. | Defects and material wear and tear. | - **Hardware** |
| 11 | Artefakte EKG. Alarmgrenzen zu eng. | ECG artifacts.  Alarm-limits set too narrow. | - **Artifacts** (ECG) - **Alarms:** alarm limit set up |
| 12 | SpO2 Artefakte. EKG-Artefakte durch unsachgemässe Ableitungspositionierung.  Alarme unterdrücken und Übersehen von neuen Zuständen  Grenzen falsch eingestellt (Durch Voroperator) | SpO2 artifacts. Artifacts of the ECG caused by improper positioning of the electrodes.  Suppressing alarms and overlooking new patient states.  Alarm-limits set incorrectly (for example, by a previous operator). | - **Artifacts** (ECG, SpO2) - **Alarms:** alarm fatigue - **Alarms:** alarm limit set up |
| 13 | Bei Spital- bzw. Monitorwechsel lange (tagelange) Angewöhnungszeit an die neuen Monitore. | When changing the hospital or the monitor type, it takes a long time (up to many days) to get used to the new monitors. | - **Human factors:** familiarization effects |
| 14 | Alarmgrenzen je nach Person unterschiedlich eng eingestellt-> alarmiert sehr schnell oder lange nicht | Alarm-limits are set differently by different people -> either the monitor then alerts very quickly or not at all for long. | - **Alarms:** alarm limit set up |
| 15 | ich verstehe nicht immer alle Zahlen | One does not always understand all the numbers. | - **Software:** information presentation - **Human factors:** human performance |
| 16 | Fehlmessungen/ Fehlalarme- den Patient mit dem Monitor vergleichen... dann eine Diagnose ableiten. | Incorrect measurements / false alarms.  Compare the patient with the monitor then derive a diagnosis. | - **Artifacts** - **Alarms:** alarm limit set up - **Comment/suggestion** |
| 17 | Antwort: Man ist sehr stark auf einen bestimmten Monitor «fixiert» und hat Mühe sich auf einem neuen Gerät zurechtzufinden. | One gets very "fixated" on a particular monitor device and has difficulty learning a new device. | - **Human factors:** familiarization effects |
| 18 | Sie sind schwer | They are heavy. | - **Hardware:** size/weight |
| 19 | Hin und wieder Suchen von Werten. | Now and then, looking for values. | - **Software:** information presentation - **Software:** interface design |
| 20 | - Nicht wissen wo was eingestellt werden kann - Anordnung der Kurven immer unterschiedlich   Wenn man sich an einen Monitor gewöhnt hat, und dieser einigermassen Sinnvoll arbeitet: KEINE | - Not knowing where specific settings can be made.  - The waveforms are often arranged differently.  Once one gets used to a monitor, and it works correctly: NO problems. | - **Software:** interface design - **Software:** information presentation - **Human factors:** familiarization effects |
| 21 | technische Probleme, Monitoring an sich zufriedenstellend. | Technical problems.  The monitoring per se is satisfactory. | - **Hardware** |
| 22 | Es sollte Patientengruppenspezifisch sein. Nach Altersgruppen oder ASA Klassen. Bei gesunden Pat. toleriert man eventuell z.B. etwas mehr Hypotonie/Hypertonie als bei Vorerkrankten Patienten.  Zudem sind wir an Zahlen gewöhnt, es dauert einige Zeit, bis man schnell Situationen einschätzen kann. Besonders für die ältere Generation kann es schwierig werden. | The monitoring should be patient group specific, for example by age group or ASA class. For example, in healthy patients, one may tolerate more hypotension/hypertension than in patients with pre-existing conditions.  Besides, we are used to numbers. It takes some time until one can quickly assess a whole situation. Especially for the older generation, this can be difficult. | - **Comment/suggestion** - **Human factors:** human performance |
| 23 | Zu schwer. Wireless wäre interessant. Wunschvorstellung: 1 Device am Patienten anbringen für alle wichtigen Vitalparameter. Zuviele individuelle Einstellungen. Einfaches Handling der Displaylayoutsmodifikationen (Intuitiv a la Apple). | Monitors are too heavy.  Wireless would be interesting. A wish: a single device on the patient, which measures all vital-signs.  Monitors have too many individual settings.  The screen layout should be easily adjustable (intuitive as Apple products). | - **Hardware:** size/weight - **Hardware: cables** (wireless) - **Software:** interface design - **Software:** intuitiveness |
| 24 | Umstand, dass mehrere Monitore nötig sind; teils umständliche Alarmierung settings; | The fact that several monitors are needed to get all of the vital sign information.  Cumbersome alarms  Settings. | - **Software:** information presentation - **Alarms:** alarm fatigue - **Software:** interface design |
| 25 | Technische Probleme (Zb EKG-Elektroden). Zuviele Alarme überdecken den «wichtigen» Alarm. | Technical problems (e.g., ECG electrodes).  Too many alarms mask the "important" alarm. | - **Hardware: cables** (ECG) - **Alarms:** false alarms |
| 26 | Schlechte Sicht auf den Monitor  Falsch/ schlecht eingestellte Alarme  Zuwenig Aufmerksamkeit für den Monitor | An inadequate view of the monitor.  Wrong or poorly set alarms.  One pays too little attention to the monitor. | - **System factors:** work environment - **Alarms:** alarm limit set up - **Human factors:** human performance |
| 27 | Parameterprioritäten nicht normiert. Darstellung/Normgrenzen nicht einheitlich. Bessere Standarteinstellung erwünscht. Nicht intuitive Menuführung. Umständliche Kabel beim Patiententransport (zB besser bei System «Philips») | Vital sign priorities not normalized.  Presentation / standard alarm-limits not uniform.  Better standard setting desired.  Menu navigation non-intuitive.  Cables cumbersome when transporting patients (better with the "Philips" system). | - **System factors:** lack of standardization - **Alarms:** default settings - **Software:** interface design - **Cables** |
| 28 | Alarme und Alarmeinstellungen, farbliche Kodierung der Alarme oft nicht gut | Alarms and alarm settings.  Color coding of the alarms often not good. | - **Alarms:** alarm limit set up - **Software:** information presentation |
| 29 | EKG-Ableitungen «applicator-dependant», EKG-Störsignale. Art, lage und Katheter abhängig, Nievaue bei lagerung Änderung bzw BD änderungen | (Function of) ECG leads «user dependent.»  ECG interferences.  Type, location, and catheter dependent.  Level (of blood pressure transducer) when the patient position is changed, or the blood pressure changes. | - **Hardware: cables** material (ECG) - **Artifacts** (ECG, BP) |
| 30 | Diskriminierung von Artefakten und echten Problemen. | Discrimination between artifacts and real problems is difficult. | - **Artifacts** |
| 31 | Artefakte und Fehlalarme.  Fehlalarme | Artifacts and false alarms. | - **Artifacts** - **Alarms:** false alarms |
| 32 | - Technische Artefakte und Fehlalarme - Nicht intuitives Handling | - Technical artifacts and false alarms.  - Non-intuitive operation. | - **Artifacts** - **Alarms:** false alarms - **Software/Hardware:** intuitiveness |
| 33 | Kabel umständlich->wireless. Keine klaren Standardeinstellungen. | Cable cumbersome-> wireless.  No clear default settings. | - **Hardware: cables** (wireless) - **Alarms:** default settings - **System factors:** lack of standardization |
| 34 | Zuviele Informationen auf dem Monitor können evtl vom Patienten ablenken. Alarme können irreführend sein. | Too much information on the monitor may distract from the patient.  Alarms can be misleading. | - **Software:** information presentation - **Human factors:** human performance - **Alarms:** false alarms |
| 35 | zu kleine Displays -> zu kleine Darstellung der Informationen | Displays too small -> presentation of the information too small. | - **Hardware:** display - **Software:** information presentation |
| 36 | kleiner Display mit schlechter Auflösung. Zu klobig, zu schwer, Display nicht seperat abnehmbar. Touchscreen wäre wohl auch besser bzw vor allem intuitiver. | Small display with reduced resolution.  Too clumsy, too heavy.  Display not separately removable.  Touchscreen would probably be better or above all more intuitive. | - **Hardware:** display (touchscreen) - **Hardware:** size/weight |
| 37 | Viel zu schwer, zuviele Kabel | Too heavy.  Too many cables. | - **Hardware:** size/weight - **Cables** |
| 38 | Alarme nerven. | Annoying alarms. | - **Alarms:** alarm fatigue |
| 39 | Zu schwer. Wire-less wäre angenehm. | Too heavy.  Wireless would be nice. | - **Hardware:** size/weight - **Hardware: cables** (wireless) |
| 40 | Ich finde unsere Monitore generell gut und habe (finde ich jedenfalls) wenig Mühe, mir in kurzer Zeit einen Ueberblick zu verschaffen. Am ehesten «durch die Lappen» geht in gewissen Fällen zum Beispiel die ST-Strecken-Ueberwachung oder generell EKG-Veränderung (die Morphologie betreffend, nicht die Rhythmik). | Our monitors are generally good, and I have little trouble getting an overview in a short time.  Most likely, the ST segment monitoring or generally ECG changes are overlooked (the morphology, not the rhythm). | - **Software:** information presentation - **Human factors:** human performance |
| 41 | Beatmungsparameter und Kreislaufparameter auf zwei Displays. Displays bei vielen Parametern teilweise unübersichtlich / zu klein.  Antwort: teilweise schwer zu transportieren, kleinere Transportmonitore wären besser  Alarmgrenzen müssen teilweise erst angepasst werden, gerade wenn der Monitor aus dem Aufwachraum zurück kommt | Ventilation vital-signs and circulatory vital-signs on separate displays.  Displays with many vital-signs are partly confusing / too small.  Sometimes difficult to transport, smaller transport monitors would be better.  Alarm-limits sometimes have to be adjusted just when the monitor comes back from the recovery room. | - **Software:** information presentation - **Hardware:** display - **Hardware:** size/weight - **Alarms:** alarm limit set up |
| 42 | Zwei Displays. Zuviele Alarmtönen die ggfs de-sensibilisieren. | Two displays.  Too many alarm sounds are desensitizing. | - **Software:** information presentation - **Alarms:** alarm fatigue |
| 43 | ST Strecken-Beurteilung nicht ganz einfach. Potentiell zu viele Parameter auf dem Bildschirm (zuviele Information auf einem Bild). | ST assessment not easy.  Potentially too many vital-signs on the screen (too much information on a picture). | - **Software:** information presentation - **Human factors:** human performance |
| 44 | Schwere Monitore, Kabelsalat, Kurze Akkulaufzeit, Einstellungen nicht intuitiv, Nicht übersichtlich gestaltet (zuviele Tasten) | Heavy Monitors.  Cable-clutter.  Short battery life.  Setup not intuitive.  Not arranged clearly (too many keys). | - **Hardware:** size/weight - **Hardware: cables** (cable clutter) - **Hardware:** components (battery, keys) - **Software:** intuitiveness - **Software:** information presentation |
| 45 | Keine klare Standardkonfiguration. | No clear standard configuration. | - **Alarms:** default settings - **System factors:** lack of standardization |
| 46 | Zu kleine Zahlen, von weitem nicht gut sichtbar. | Numbers too small, not visible from a distance. | - **Hardware:** display - **Software:** information presentation |
| 47 | viele Alarme, zu viele Klicks bis die Alarmgrenzen so gestellt sind, wie man will. Zu viele Klicks bis Monitor-Konfiguration so ist, wie man es will.  Super Tools teilweise sehr weit in Unter-Menü versteckt, so dass ich sie nie finde... | Many alarms, too many clicks needed to set the alarm-limits.  Too many clicks needed to configure the monitor. Great tools hidden in submenus, so they are hard to find. | - **Alarms:** false alarms - **Alarms:** alarm limit set up - **Software:** interface design |
| 48 | -zu viele Zahlen pro Display  -wichtige Parameter zu wenig hervorstechend  Häufig falsche Alarme führen zum Ignorieren dieser. | Too many numbers per display.  - critical vital-signs too little prominent  Frequent false alarms lead to ignoring of alarms. | - **Software:** information presentation - **Alarms:** false alarms - **Alarms:** alarm fatigue |
| 49 | viele Informationen visueller wie auditiver Art, Blick für Relevantes geht verloren | Much visual and auditory information, the sense for the relevant gets lost. | - **Software:** information presentation |
| 50 | zu viel verlassen auf die Technik. | One relies too much on technology. | - **Comment/suggestion** |
| 51 | unnötige Alarme bei fixen Grenzen   - Uniformität der akustischen Alarmen (eine Abstufung mehr) | Unnecessary alarms set at fixed limits.  Acoustic alarms are uniform (one more subdivision). | - **Alarms:** alarm limit set up - **Comment/suggestion** |
| 52 | Kabel nicht sehr widerstandsfähig/robust. Schwerer Monitor. Kabelsalat (1 Masterkabel?). | Cable not very resistant/robust.  Heavy monitor.  Cable-clutter (1 master cable?). | - **Hardware: cables** - **Hardware:** size/weight - **Hardware: cables (**cable clutter) |
| 53 | Gewicht zu gross, Drehknopf aus dem letzten Jahrhundert. Kabel. Zu viele Kabel. Immer. | Too heavy.  Knob from the last century.  Too many cables. Always. | - **Hardware:** size/weight - **Hardware:** components (knob) - **Hardware:** cables |
| 54 | Ein Mal gemessene Werte bei nicht kontinuierlicher Datenaufnahme (z.B. Blutdruck) sollte nach einer gewissen Zeit (z.B. 3 oder 5 Minuten) wieder verschwinden. | Measured values for non-continuous data collection (e.g., blood pressure) should disappear after a specific time (e.g., 3 or 5 minutes). | - **Comment/suggestion** |
| 55 | viele unterschiedliche Bildschirme. Gleiche Info oft an unterschiedlichem Ort/Farbe/Grösse. Kabel. | Many different screens.  Same info often at different location / color / size.  Cables. | - **Software:** information presentation - **Cables** |
| 56 | Batterien oft leer.  Nur begrenzte Parameterzahlen darstellbar, oft umständliche Umstellung der dargestellten Parameter. | Batteries often empty.  Only a limited number of vital-signs can be displayed on one screen.  Setting up different vital-signs is often laborious. | - **Hardware:** components (battery) - **Software:** information presentation - **Software:** interface design |
| 57 | Technische Defekte -> zu wenig robust | Technical defects -> not robust. | - **Hardware** |
| 58 | Viel Info für schnelle Uebersicht, Alarmgrenzen nötig  Es sollte immer die gleiche Bild- und Farbzusammenstellung sein, ist aber häufig nicht so, je nach dem von welcher Abteilung der Monitor kommt. | Too much information to get a quick overview, alarm-limits required.  It should always be the same layout and color combination, but it is often not so, depending on the department that the monitor originates from. | - **Software:** information presentation - **Alarms:** alarm limit set up - **Software:** information presentation - **System factors:** lack of standardization |
| 59 | Zahlen vergessen bis Sie auf dem Protokoll einzutragen sind  Kabelsalat. Nicht intuitive Bedienung. | Values are forgotten before they can be written to the log.  Cable-clutter.  Non-intuitive operation. | - **Comment/suggestion** - **Human factors:** human performance - **Hardware: cables** (cable clutter) - **Software:** intuitiveness |
| 60 | Kabelsalat. Zu viele Informationen (bSp Gasparameter) | Cable-clutter.  Too much information (e.g. anesthesia gas values from the respirator) | - **Hardware: cables** (cable clutter) - **Software:** information presentation |
| 61 | Kabelsalat, schwere, grosse Apparate  Spannend wär ein stilles übersichtliches Monitoring für zB Rega, Angio..... | Cable-clutter.  Heavy.  Big appliances.  Exciting would be quiet, clear monitoring for example for Rega (helicopter emergency service), and Angio (where, in our hospital, a former operating surgeon did not tolerate sound disturbance). | - **Hardware: cables** (cable clutter) - **Hardware:** size/weight - **Comment/suggestion** |
| 62 | Übersehen von pathologischen Werten. Zuviele nummerische Informationen | Failing to notice pathological values.  Too much numerical information. | - **Human factors:** human performance - **Software:** information presentation |
| 63 | Zu wenig robust für den Alltagsdurchlauf (viel Verschleissmaterial), zu schwer, zu viele einzelne Kabel. | Not robust enough for the everyday run (much wear material).  Too heavy.  Too many individual cables. | - **Hardware:** components - **Hardware:** size/weight - **Cables** |
| 64 | Kabelsalat. Technische Defekte der Kabel. | Cable-clutter.  The cables have technical defects. | - **Hardware: cables (**cable clutter) - **Hardware: cables** |
| 65 | Es werden Werte angezeigt, die ich nicht brauche und/oder nicht interpretieren kann  Diskrepanz zum Beispiel CO2-Messung (%, kPa)  Ständige Alarme bei Elektrokauter und EKG -> Rhythmusalarm  2min Alarm-Aus-Knopf sollte nur den Alarm ausschalten bei welchem der Knopf gedrückt wurde  schlechte Grundeinstellung von Alarmgrenzen | I do not need or cannot interpret some of the values that are displayed. For example, CO2 measurement (%, kPa).  Constant alarms for electrocautery and ECG -> rhythm alarm.  The 2min alarm-off button should only turn off the alarm for which the button was pressed.  The default setting of the alarm-limits is inadequate. | - **Software:** information presentation - **Human factors:** human performance - **Artifacts** (ECG) - **Comment/suggestion** - **Alarms:** default settings - **Alarms:** alarm limit set up |
| 66 | Ableitungsprobleme bei EKG, Artefakte, Kleber fallen ab. Kauterartefakte im EKG. Kabeldefekte. Blutdruckmessung RR wird durch Chirurg der abstützt verfälscht. | ECG lead problems.  Artifacts.  ECG conductive adhesives fall off.  Cautery artifacts on the ECG.  Cable defects.  Blood pressure measurement RR gets distorted when a surgeon leans on it with his body weight. | - **Hardware: cables** (ECG) - **Artifacts** (ECG) - **Comment/suggestion** |
| 67 | Schwer, Kabelsalat, Bedienung nicht intuitiv. | Heavy.  Cable-clutter.  Operation non-intuitive. | - **Hardware:** size/weight - **Hardware: cables** (cable clutter) - **Software:** intuitiveness |
| 68 | Zu sensibel. Alarmt zu häufig müssen zu häufig eingestellt werden. Alarmgrenzen müssen ständig angepasst werden. | Too sensitive. It warns too often.  One must set and adjust alarm-limits too frequently. | - **Alarms:** false alarms - **Alarms:** alarm limit set up |
| 69 | Warum haben wir keinen Touchscreen? | Why do we not have a touchscreen? | - **Hardware:** display (touchscreen) |
| 70 | ungeignete Basiskonfiguration | The basic configuration is unsuitable. | - **Alarms:** default settings |
| 71 | Einstellung der gewünschten Parameter so, dass man auf einen Blick alles übersichtlich sieht.  Alarmgrenzen unpraktisch voreingestellt.  Mühsames «Heranholen» der Trendgrafik/-tabelle und der letzten Werte.  Finden des richtigen Bildes oder Untermenüs. Suchen und Akrtivieren der gewünschten Parameter («auf welchem Kanal ist die Arterie jetzt nochmal eingesteckt und wo kann ich den auswählen?»). Bei ungewohnten Monitoren ist der Patient effektiv schlechter einegstellt von den Votalparametern her, da sie viel schlechter und langsamer wahrgenommen werden. | Setup of the desired vital-signs so that everything is visible at a glance.  Impractical default alarm-limits.  Effortless "catch up" with the trend graph/table and the last values.  Find the right image or submenu. Find and adjust the desired vital-signs ("Which channel is the artery on again?")  The finding of the right image or submenu is difficult.  In unfamiliar monitors, the patient is effectively worse off due as the vital-signs are perceived much worse and slower. | - **Software:** interface design - **Alarms:** alarm limit set up - **Alarms:** default settings - **Comment/suggestion** |
| 72 | Personalisieren der präferierten Einstellungen nicht intuitiv/resp umständlich. | Personalization of the preferred settings is non-intuitive/laborious. | - **Software:** interface design - **Software:** intuitiveness |
| 73 | Die «falschen» Alarme.  Alle wichtigen Alarmtöne sind gleich. | The alarms are "wrong."  All critical alarm tones sound the same. | - **Alarms:** false alarms - Comment/suggestion |
| 74 | Akku, Wackelkontake, fehlende Kabel, kein Touchscreen beim Drägher Monitoring, keine intuitive Bedienbarkeit | Battery, loose contacts, missing cables, no touchscreen. The operation of the Dräger Monitoring is non-intuitive. | - **Hardware:** components (battery, contacts) - **Hardware:** display (touchscreen) - **Cables** - **Software:** intuitiveness |
| 75 | Bei langen Ops kommt es vor, dass der langsame Sättigungsabfall nicht bemerkt wird (zBsp Bariatrie und Atelektasenbildung). ST Strecke wird teilweise nicht ernst genommen/beobachtet von Anfang bis Ende. Air trapping wird nicht durch Respi alarmiert.  Alarme müssen korrekt eingestellt werden um hilfreich zu sein.  Bei fehlendem Training werden Alarme ggfs nicht richtig eingeordnet. dH jedes noch so weit entwickelte Monitoring benötigt erfahrung und Lernzeit. | During extended operations, the slow saturation decline (e.g., bariatric surgery and atelectasis formation) is sometimes not noticed.  The ST-segment is sometimes not taken seriously / observed from beginning to end.  The respirator does not alert in case of air trapping.  Alarms must be set correctly to be helpful.  If there is no training, one cannot interpret alarms correctly, meaning that even the most advanced monitoring requires experience and learning time. | - **Human factors:** human performance - **Comment/suggestion** - **Alarms:** alarm limit set up |
| 76 | Material und Länge der verschiedenen Kabel (verhängen sich gerne), Gewicht, Batteriedauer, Transportierfähigkeit, | Material and length of the different cables (getting tangled easily).  Weight.  Battery life.  Transportability. | - **Hardware: cables** (cable clutter) - **Hardware:** size/weight - **Hardware:** components (battery) |
| 77 | Nicht funktionierendes Monitoring (defekte Kabel, Hämopot, Sättigungsmessung), fehlendes Material (Arbeitsabläufe nicht optimal?) | Malfunctions of the monitoring: defective cables, hemopot (Dräger), saturation measurement.  Missing material (work processes not optimal?). | - **Hardware: cables** - **Hardware:** components (Transducer, SpO2) - **Comment/suggestion** - **System factors:** lack of standardization |
| 78 | Kein Touchscreen (Drehrad), Bedienung nicht intuitiv, viele Zwischenschritte im Bedienungsmenü bis man am gewünschten Ort ist (Zuviele Unterordner). | No touchscreen (rotary knob).  Operation non-intuitive.  Many intermediate steps in the menu until one gets to the desired location (too many subfolders). | - **Hardware:** display (touchscreen) - **Software:** intuitiveness - **Software:** interface design |
| 79 | Unzuverlässige Akkus. Materialprobleme (technische Defekte: SpO2-Messung, RR-Messung). Artefakte. Schweres Equipment. | Unreliable batteries.  Material problems.  Technical defects: SpO2 measurement, RR measurement.  Artifacts.  Heavy equipment. | - **Hardware:** components (batteries, SpO2, BP) - **Artifacts** - **Hardware:** size/weight |
| 80 | Kabel sind störend  Monitoring störungsanfällig  Klinische Überwachung geht vor lauter Monitoring zu stark in den Hintergrund/vergessen. Viele können einen Patienten nicht mehr klinisch Überwachen. | Cables are disturbing.  Monitoring is prone to interference.  Too much data overshadows clinical monitoring. Many can no longer clinically monitor a patient. | - **Cables** - **Artifacts** - **Software:** information presentation - **Comment/suggestion** |
| 81 | - Zu viele Daten/Werte auf dem Monitor - Manche Daten sind teilweise unnütz | - Too much data/values on the monitor.  - Some data is partly useless. | - **Software:** information presentation - **Comment/suggestion** |
| 82 | - Falsch eingestellte Alarmgrenzen (Eichung…..) - Ein pathologischer Wert steht zwar auf dem Monitor, der Überwacher hat ihn aber nicht realisiert, da eine Zahl an sich ja nicht auffällt. - Ständig nervige Alarme, welche einfach weg gedrückt werden | - Inadequately set alarm-limits (calibration).  Although a pathological value is on the monitor, the user does not recognize it because a number is not readily perceptible.  - Constantly annoying alarms get merely pushed away. | - **Alarms:** alarm limit set up - **Human factors:** human performance - **Alarm fatigue** |
| 83 | Zuviele Alarme. Grundeinstellungen sind von Monitor zu Monitor variabel (und keine Standard). Bedienung nicht intuitiv. | Too many alarms.  Basic settings are variable from monitor to monitor (and not standard).  The operation is non-intuitive. | - **Alarms:** false alarms - **System factors:** lack of standardization - **Software:** intuitiveness |
| 84 | Zuviel Alarme z.B. bei Evita geht der wirklich relevante Alarm unter.  Zuviel Monitoring am Monitoring und zu wenig Monitoring am Patienten. Klinisches Monitoring in Betracht ziehen. | Too many alarms. With the Evita (Dräger) the critical alarm is not perceived.  Too much monitoring of monitoring itself and too little monitoring of the patient. Consider clinical monitoring. | - **Alarms:** false alarms - **Comment/suggestion** |
| 85 | Verschiedene geographische Lokalisationen der verschiedenen Informationsquellen (Relaxometrie, Respiratorparameter, restliche Parameter) | Different geographic locations of the different sources of information (relaxometry, respiratory vital-signs, other vital-signs). | - **Comment/suggestion** |
| 86 | Im Alltag zu viele Alarme die bestätigt werden müssen, resp vom wichtigen Alarm ablenken. (Vielleicht ein «Einleiten»button). Monitore kommen teilweise mit «alle Alarme aus» zurück und pathologische Werte werden nicht gemeldet. | In everyday life, too many alarms must be confirmed, which distract from the critical alarm. (Maybe an "induction" button).  Monitors sometimes come back with the setting "all alarms off," and pathological values are not perceived. | - **Alarms:** false alarms - **Alarms:** alarm fatigue - **Alarms:** alarm limit set up |
| 87 | schlechte (Standard)Alarmgrenzen (nicht immer sinnvoll), Neigung zum Alarmstumm-Knopf | Poor (default) alarm-limits (not always meaningful), tendency to alarm mute button. | - **Alarms:** default settings - **Alarms:** alarm limit set up - **Alarms:** alarm fatigue |
| 88 | Unterschiedliche Voreinstellungen der Alarmgrenzen, abgeschaltete Alarme in anderen Räumlichkeiten, unterschiedliche Narkoseführende haben unterschiedliche Alarmgrenzen | Different default settings of the alarm-limits.  In some areas, some alarms get deactivated.  Different anesthetists have different alarm-limits. | - **Alarms:** default settings - **Alarms:** alarm limit set up - **System factors:** lack of standardization |
| 89 | Uebersichtlichkeit bei vielen gemessenen Parametern, v.a. bei Ueberlagerungen. Verlaufsanzeigen (Trend). | Surveillance when many vital-signs are measured, especially when superimposed over one another. Longitudinal indicators (trends). | - **Software:** information presentation |
| 90 | schwer | heavy | - **Hardware:** size/weight |
| 91 | Unübersichtlich, zuviele Zahlen,  Häufig zuviel und zu schnell ausgelöste Alarme 🡪 führt zu Desensibilisierung  Standardeinstellungen sind zu laut. | Confusing, too many numbers.  Alarms are often triggered too much and too fast, which leads to desensitization.  Default settings are too loud. | - **Software:** information presentation - **Alarms:** false alarms - **Alarm**s: alarm fatigue - **Alarms:** default settings |
| 92 | zu viele «unwichtige» alarme | Too many "unimportant" alarms. | - **Alarms:** false alarms |
| 93 | Zu schwer. Touchscreen erwünscht. Wireless. | Too heavy.  Touchscreen desired.  Wireless. | - **Hardware:** size/weight - **Hardware:** display (touchscreen) - **Hardware: cables** (wireless) |
| 94 | Bedienung. technische Schwierigkeiten. Kabel defekt oder Einstellung?  Monitor schwer. | Operation.  Technical problems. Is the cable defective or is something incorrectly set?  Monitor heavy. | - **Software:** interface design - **Hardware: cables** - **Hardware:** size/weight |
| 95 | müde 🡪 muss mehrfach hinsehen bis Information ankommt – Visual Patient könnte hier hilfreich sein | Tired: One has to look several times until the information arrives - Visual Patient could be helpful here. | - **Human factors:** human performance - **Software**: information presentation |
| 96 | technische Probleme z.b. EKG Artefakte, z.b. arterielle Blutdruckemssung (Druckbeutel insuffizient), SpO2 Messung anfällig, da Clip auch misst wenn quer am Finger, Sättigung dann aber häufig falsch tief. | Technical problems, e.g., ECG artifacts, arterial blood pressure bag insufficient filled, SpO2 measurement prone for error, as clip also measures when transversely on the finger. However, saturation is often incorrectly low in this case. | - **Artifacts** (ECG, BP, SpO2) - **Comment/suggestion** |
| 97 | Lichtverhältnisse und Winkel zum Monitor | Lighting conditions and viewing angle to the monitor. | - **System factors:** work environment - **Hardware:** Display |
| 98 | Alarmgrenzen müssen individuell eingestellt werden. Bedienung nicht intuitiv.  Schwer. Unzuverlässige Akkurestlaufzeit. | Alarm-limits must be set individually.  Operation is non-intuitive.  Heavy.  Unreliable battery life. | - **Alarms:** alarm limit set up - **Usability:** intuitiveness - **Hardware:** size/weight - **Hardware:** components (battery) |
| 99 | Artefakte, ständige Alarme die zu „Alarmermüdung“ führen d.h. Alarme werden einfach weggeklickt | Artifacts.  Constant alarms leading to "alarm fatigue," i.e., alarms are merely clicked away. | - **Artifacts** - **Alarms:** alarm fatigue |
| 100 | Konzentration auf alle Parameter gleichzeitig ist manchmal schwierig. | Concentrating on all vital-signs at the same time is sometimes difficult. | - **Human factors:** human performance - **Software:** information presentation |
| 101 | Alarmoverkill (also drei blinkende Alarme, welcher ist prioritär?)  Systematisches Scannen und Erfassen von Prioritäten muss man trainineren, hierfür braucht es methoden, welche dann aus dem Ruder laufen, wenn jemand einen Bildschirm umstellt (z.B. weisse NIBP Werte vs. weisse Atemfrequenz.  Alarmqualitäten – der Dräger hat einen gelben und einen roten, akkustisch getrennt. Die blöde Evita Transportrespikrücke hat einen Alarm für alles – hey, ich hab einen Akku oder hey, Dein Patient schnauft nicht. | Alarm overkill (e.g., three flashing alarms, which one is most important?)  Systematic scanning and prioritization must be trained, and this requires methods that get out of hand when someone adjusts a screen differently (for example, white NIBP values vs. white respiratory rate).  Alarm qualities - the Dräger (Infinity) monitor has a yellow and a red alarm, acoustically separated. The Dräger Evita Monitor has a single alarm for everything. - "Hey, is there a low battery, or hey, is the patient not ventilated?" | - **Alarms:** alarm fatigue - **Human factors:** human performance - **Software:** information presentation - **Comment/suggestion** |
| 102 | Sind schwer.  Blick muss für eine umfassende Zustandseinschätzung über mehrere Monitore und Zahlen wandern, welche dann interpretiert werden müssen. | The monitors are heavy.  For a comprehensive state assessment, the gaze must travel across multiple monitors and numbers, which must then be interpreted. | - **Hardware:** size/weight - **Software:** information presentation |
| 103 | Kabelsalat -> Kabelloses Monitoring  Unzuverlässige Werte bei Zb kalter, schweissiger Haut des Patienten  EKG Elektrodenplatzierung bei morbid adipösen/kachektischen Patienten und spezieller Lagerung  Unergonomische Arbeitsplätze  MRT Monitoring: nicht alltagstaugliche Hardware (Pulsoxy, EKG-Artefakte) | Cable-clutter -> Wireless Monitoring.  Values become unreliable with cold, sweaty skin of the patient.  ECG electrode placement in morbidly obese/cachectic patients and with particular patient positioning.  Unergonomic working environment.  MRI monitoring: hardware that is not suitable for everyday use (pulse oximeter, ECG artifacts). | - **Hardware: cables** (cable clutter) - **Hardware: cables** (wireless) - **Comment/suggestion** - **System factors:** work environment |
| 104 | TOF sollte auf dem Monitor sein. | TOF (Train-of-four relaxometry monitoring) should be on the monitor. | - **Comment/suggestion** |
| 105 | Kabelsalat, Defekt, schwär | Cable-clutter.  Defects.  Weight. | - **Hardware: cables** (cable clutter) - **Hardware:** material - **Hardware:** size/weight |
| 106 | Immer Kabelsalat. | Always cable-clutter | - **Hardware: cables** (cable clutter) |
| 107 | alarmgrenzen einstellen ? passiert häufig nicht. Wie stelle ich patientenadaptierte alarmgrenzen ein (am Modell)  Artefakte abgrenzen. | Alarm-limits set? Often it does not happen.  How can patient-adapted alarm-limits be set on different models?  Distinguish artifacts from reality. | - **Alarms:** alarm limit set up - **Software:** interface design - **Artifacts** |
| 108 | Kabel dekonnektiert/falsche Werte abgenommen. Unübersichtliche Darstellung, zu viele Farben (haben Farben einen Sinn/eine Aussage). Gelegentlich schwierig einsehbar, welche Alarme bzw. Alarmgrenzen eingestellt sind. | Cable disconnected / incorrect values measured.  Unclear presentation, too many colors (do colors have meaning / a statement?).  Occasionally it is difficult to see which alarms or alarm-limits have been set. | - **Hardware: cables** (connections) - **Software:** information presentation - **Software:** interface design |
| 109 | Kabelsalat, Fehlfunktionen, Darstellung, EKG Ableitungen, Artefakte | Cable-clutter.  Malfunctions.  Presentation.  ECG leads.  Artifacts. | - **Hardware: cables (**cable clutter) - **Hardware** - **Software:** information presentation - **Hardware: cables** (ECG) - **Artifacts** |
| 110 | - kein globales Monitoring vorhanden mit allen Parametern (Vitaldatenmonitoring, Beatmungsmonitoring, TOF Monitoring, etc.  - Teilweise physische Verteilung der wichtigsten Monitore im Raum.  - Bei nicht bekannten «Warntönen» wird in Stresssituationen keine Reaktion, auf diese, ausgelöst.  - Warntöne werden teilweise ignoriert. «Das ist ja immer so» oder «Alarm geht gleich wieder weg»  - Patientenmonitoring ist zu schwer (kg)  - Bedienung. Verschachtelte Menüs, kein Touchscreen. | - no global monitoring available with all vital-signs (vital data monitoring, ventilation monitoring, TOF monitoring, and so on).  - The positioning of the most critical monitors in the room is inadequate.  - Unknown "warning" alarm tones, do not trigger a reaction in stressful situations.  - Warning sounds get frequently ignored. "It is always like this" or "the alarm goes right away."  - Patient monitoring is too heavy (kg).  - Operation.  - Nested menus, no touchscreen. | - **Software:** information presentation - **System factors:** work environment - **Comment/suggestion** - **Alarms:** alarm fatigue - **Hardware:** size/weight - **Software:** interface design - **Hardware:** display (touchscreen) |
| 111 | Gewicht, Kabel. | Weight.  Cable. | - **Hardware:** size/weight - **Cables** |
| 112 | Zu viele Kabel. Schweres Equipment/Monitor. | Too many cables.  Heavy equipment/monitor. | - **Hardware: cables** - **Hardware:** size/weight |
| 113 | zuviele Alarme gleichzeitig auf die man innerhalb einer Prioritätenliste reagieren muss. | Too many alarms at the same time, which one must respond to by creating a mental priority list. | - **Alarms:** false alarms |
| 114 | Relativ grosse Fläche mit den verschiedenen Displays, die es gleichzeitig zu registrieren gibt. | A relatively large area with different displays that one must monitor continuously. | - **Software:** information presentation - **System factors:** work environment |
| 115 | Sehr schwere Monitore. Positionierung zu hoch oder zu weit weg vom Betrachter. Ansonsten zufrieden. Trendkurven sehr hilfreich und übersichtlich. | Very heavy monitors.  Positioning too high or too far away from the viewer.  Otherwise satisfied. Trend curves are very helpful and clear. | - **Hardware:** size/weight - **System factors:** work environment - **Comment/suggestion** |
| 116 | Bei Notfallsituationen muss man sich einen raschen und sicheren Überblick verschaffen, was mit dem aktuellen Monitoring nicht immer möglich ist.  ‘Rasch’ ist nicht immer das Beste, aber in Situationen mit Problemen (Anästhesie) ist das wichtig. | In emergency situations, one must get a quick and safe overview, which is not always possible with the current monitoring.  'Speed' is not always the best, but in situations with problems (anesthesia) it is essential. | - **Software:** information presentation - **Comment/suggestion** |
| 117 | Nur Kurven und Zahlen/Werte, zuviel «falsche Alarme» | Only curves and numbers/values.  Too many «false alarms.» | - **Software:** information presentation - **Alarms:** false alarms |
| 118 | Monitoreinstelllungen, Nicht immer selbe Einstellung, heisst Auge kann nicht geübt sein. | Monitor settings: Not always the same setting, that means the eye cannot get used to it. | - **System factors:** lack of standardization - **Software:** information presentation |
| 119 | Zu viele Farben; sie wirken dissuasiv.  Zu viele unnötige Alarme (bei Einleitung ist der häufigste unnötige Alarm der diast. Blutdruck, dieser wird einfach weggedrückt, ohne zu schauen, weil die wichtigen Parameter im Normbereich liegen). Konsequenz: Alarme werden nicht mehr ernst genommen. | There are too many colors; they look overwhelming.  Too many unnecessary alarms (at induction, the most common unnecessary alarm is the diastolic blood pressure, which is pushed merely away without looking because the critical vital-signs are within reasonable limits). Consequence: alarms are not taken seriously. | - **Software:** information presentation - **Alarms:** false alarms - **Alarms:** alarm fatigue |
| 120 | Alarmgrenzen nicht eingestellt -> keine Alarmierung bei grossen Veränderungen  Die einzelnen Parameter sind untereinander, sequentiell angeordnet, braucht länger Zeit, bis alle erfasst sind, als wenn alles auf einem Bild erscheint. | Alarm-limits not set -> no alarm in case of significant changes.  The individual vital-signs are arranged one below the other, sequentially. It takes more time for everything to be perceived than when everything appears on a picture. | - **Alarms:** alarm limit set up - **Software:** information presentation - **Human factors:** human performance |

**Supplementary Table 2:** The results of the word count with all words that have occurred more than five times. The dark gray shading displays the topics identified by the word count. Irrespective of singular or plural, uppercase or lowercase, the word count of these words was: alarm = 52, cable = 24, heavy = 20, alarm limits = 19, artifacts = 15, ECG=15, non-intuitive = 6. ECG = electrocardiogram.

| Word | Count |
| --- | --- |
| the | 108 |
| is | 49 |
| and | 45 |
| to | 40 |
| a | 39 |
| not | 38 |
| are | 33 |
| **alarms** | **33** |
| of | 33 |
| too | 30 |
| Too | 29 |
| monitor | 28 |
| be | 27 |
| many | 27 |
| on | 25 |
| The | 22 |
| monitoring | 20 |
| for | 19 |
| vital-signs | 19 |
| **alarm** | **19** |
| with | 17 |
| or | 17 |
| **ECG** | **15** |
| set | 15 |
| in | 15 |
| one | 14 |
| much | 13 |
| **heavy** | **13** |
| **alarm-limits** | **12** |
| patient | 12 |
| it | 12 |
| from | 12 |
| **information** | **11** |
| which | 11 |
| that | 11 |
| different | 11 |
| **Cable-clutter** | **10** |
| must | 10 |
| monitors | 10 |
| when | 10 |
| no | 10 |
| often | 9 |
| have | 9 |
| all | 9 |
| **settings** | **9** |
| time | 9 |
| values | 9 |
| problems | 9 |
| One | 9 |
| get | 9 |
| e.g | 8 |
| always | 8 |
| critical | 8 |
| gets | 8 |
| by | 8 |
| **artifacts** | **8** |
| **cables** | **8** |
| difficult | 8 |
| can | 8 |
| would | 7 |
| Technical | 7 |
| example | 7 |
| new | 7 |
| more | 7 |
| **Alarm-limits** | **7** |
| **Artifacts** | **7** |
| at | 7 |
| **Heavy** | **7** |
| sometimes | 7 |
| numbers | 7 |
| should | 7 |
| measurement | 6 |
| default | 6 |
| desired | 6 |
| used | 6 |
| right | 6 |
| **non-intuitive** | **6** |
| false | 6 |
| very | 6 |
| pressure | 6 |
| this | 6 |
| **Cable** | **6** |
| same | 5 |
| blood | 5 |
| It | 5 |
| specific | 5 |
| Many | 5 |
| away | 5 |
| everything | 5 |
| device | 5 |
| up | 5 |
| Dräger | 5 |
| Operation | 5 |
